# Positivity of contact dermatitis patch tests in an allergy and immunology dermatology service

**DOI:** 10.1186/1939-4551-8-S1-A102

**Published:** 2015-04-08

**Authors:** Kleiser Aparecida Mendes, AssunÇao De Maria Gusmao Ferreira Castro, Claudia Soido Falcao Amaral, Maria Luiza Oliva Alonso, Teresa Seiler, Monica Ribeiro Oliveira, Elizabeth Jorge Silva

**Affiliations:** 1 Instituto De Dermatologia PROF Rubem David Azulay, Brazil; 2 Hospital Universitário Clementino Fraga Filho Hucff-Ufrj, Brazil; 3 Pontifícia Universidade Católica – RJ, Brazil

## Background

Determine the prevalence of positivity to different groups of battery contact tests performed in a specialized Service.

## Methods

300 patients were evaluated at 36 months, with a diagnosis of contact dermatitis. All of them underwent delayed skin test reading (patch test), with Brazilian Standard Battery of 30 substances. The test steps (placing two readings and grading of positive responses) were performed according to the standards established by the Brazilian Study Group of Contact Dermatitis (BSGCD) and the International Contact Dermatitis Research Group (ICDRG).

## Results

Of the 300 tests, positivity was observed in 194 (65%). 106 (35%) tests were negative and characterized contact dermatitis by primary irritant. There was a predominance of females (76%) compared to males (24%). The most affected locations were hands (palms and back - 64%), feet (back and plants - 41%), arms (31%) and face (26%). Positive results were obtained by groups of substances: Anthraquinone (0.5%), Balsam of Peru (5.1%), PPD mix (7.7%), Hydroquinone (2%), Potassium bichromate (19%) Propylene glycol (1.5%), p-tertiary Butyl Phenol (1%), Neomycin (4.1%), Irgasan (1.5%), Kathon CG (10.8%), Cobalt chloride (9.2 %), Lanolin (1.5%), Thiuram mix (3.6%), Ethylenediamine (3.6%) Perfume mix (6.7%), Mercaptobenzothiazole (mix) (2%), Benzocaine (3.6%) Quaternium 15 (1.5%), Quinoline mix (6.7%), Nitrofurazone (4.6%) Paraben mix (6.1%), Epoxy-resin (1.5%), Thimerosal (24. 2%) Turpentine (2%), Carba mix (7.2%), Promethazine (7.7%), Nickel sulfate (45.8%), Colophony (5.1%), Paraphenylenediamine (12.3%), Formaldehyde (6.7%).

## Conclusions

Although positivity was observed in all groups, there was a predominance of Nickel sulfate, followed by Thimerosal and Potassium bichromate. This highlighted the importance of these substances in cases of allergic contact dermatitis admitted in our Service.

